# Minimally processed, human‐grade diet alters canine epigenetic ageing: Results from a pilot study

**DOI:** 10.1002/vro2.70047

**Published:** 2026-08-05

**Authors:** Aimee Chen, Wei Guo, Janina A. Krumbeck, Dan Su, Vincent Michels, Xiaojing Yang

**Affiliations:** ^1^ Zymo Research Corporation Irvine California USA; ^2^ MiDOG LLC Tustin California USA; ^3^ JustFoodForDogs Tustin California USA

**Keywords:** ageing, canine, DNA methylation, epigenetic age, epigenetics, kibble diet, minimally processed diet

## Abstract

**Background:**

Diet plays a critical role in promoting wellness and longevity in both humans and canines. Epigenetic ageing clocks have emerged as accurate tools for assessing biological ageing and evaluating dietary and lifestyle interventions in humans and mice.

**Methods:**

A dog‐specific epigenetic ageing clock was developed and validated using buccal swab samples from domestic dogs aged 1.5 and 17 years. The clock exhibited a strong correlation with chronological age in both the training set (*r* = 0.99) and the validation set (*r* = 0.83). To investigate the impact of diet on epigenetic ageing, purebred pugs were assigned to either one group fed a minimally processed, human‐grade food (MPHGF, *n* = 18 completed) or a processed, extruded dry kibble (EDK, *n* = 7 completed). Epigenetic age was assessed from buccal swabs collected at baseline and 6‐month timepoints. Each dog served as its own control by calculating delta age (epigenetic age minus chronological age) over the study period.

**Results:**

Dogs fed with MPHGF diet exhibited a significant reduction in delta age, decreasing from 2.75 to 1.80 years over 6 months (*p* = 0.02), indicating a trend of slowed epigenetic ageing. In contrast, dogs fed with EDK diet exhibited no significant change in epigenetic ageing (*p* = 0.86). After sex‐stratified analysis, females in the MPHGF group showed younger delta age at M6 than M0; however, not insignificant comparing to females in the EDK group.

**Conclusion:**

This study demonstrates the potential for dietary interventions to influence epigenetic ageing in dogs and provides a practical, quantitative framework for investigating healthy ageing in companion animals.

## INTRODUCTION

Although ageing is one of the most significant risk factors for disease progression and mortality in humans,^1^ ageing research has consistently demonstrated that chronological age alone does not adequately capture interindividual variation in biological ageing, disease susceptibility, health span or lifespan. Individuals of the same chronological age can exhibit markedly different physiological functions, molecular ageing profiles and risks of age‐related disease and mortality.^2^ Consequently, DNA methylation‐based estimates of epigenetic age have emerged as a quantitative framework for assessing biological ageing beyond chronological age and identifying factors associated with accelerated or delayed ageing.^3^ DNA methylation is one of the most extensively studied epigenetic modifications. It plays fundamental roles in regulating gene expression and maintaining genome stability,^4^ and during embryonic development.^5^, ^6^, ^7^ Although DNA methylation patterns are relatively stable, they remain dynamic throughout life and can be influenced by environmental factors, including diet, exposure to toxins, stress, disease and lifestyle. Consequently, alterations in DNA methylation can affect gene regulation and have been associated with biological ageing and an increased risk of age‐related diseases, including cancer and metabolic disorders.^8^, ^9^, ^10^ These age‐associated changes have established DNA methylation as a key molecular hallmark of ageing and enabled the development of epigenetic clocks that estimate biological age with remarkable accuracy.^11^

The Horvath Clock was one of the first DNA methylation‐based epigenetic ageing clocks and has paved the way for the development of numerous subsequent epigenetic ageing biomarkers.^12^ To date, the Horvath, Hannum, PhenoAge, DunedinPACE and other epigenetic ageing clocks have been used to obtain a more accurate assessment of the health‐related ageing process than chronological age alone.^12^, ^13^ Numerous studies using epigenetic age as a quantitative measure of biological ageing have demonstrated that lifestyle and environmental factors can accelerate or decelerate the ageing process in humans.^14^ Based on these accumulated scientific findings, many direct‐to‐consumer epigenetic ageing tests are now available to assess ageing in humans.^3^

As in humans, both genetic and environmental factors influence canine ageing and longevity.^15^ For example, larger dog breeds typically have shorter lifespans than smaller breeds, a phenomenon that is influenced in part by genetic variants in the insulin‐like growth factor‐1 signalling pathway.^16^, ^17^ However, inherited genetic factors alone cannot fully explain the substantial difference in health span observed among dogs of similar breed and age. Furthermore, relatively little research has investigated how epigenetic factors (e.g., diet and obesity status) potentially affect accelerated ageing and lifespan in dogs.^18^ Research into canine ageing has traditionally relied on long‐term observational studies, which provide valuable longitudinal insights but are often time‐ and resource‐intensive to conduct.^19^ Alternatively, retrospective studies leveraging electronic health records have enabled the inclusion of large canine populations.^20^, ^21^, ^22^, ^23^ However, the integration of recent molecular approaches, such as DNA methylation‐based epigenetic clocks, into canine health and ageing research remains limited. To address this challenge, several groups have developed DNA methylation‐based epigenetic clocks as convenient and quantitative molecular‐based approaches for assessing longevity in dogs.^24^, ^25^, ^26^, ^27^

In the present study, we aimed to develop and validate a canine‐specific epigenetic ageing clock based on DNA methylation status, using a non‐invasive sampling method suitable for at‐home collection by pet owners. The clock was subsequently applied in a feeding study to evaluate the effects of a minimally processed, human‐grade food (MPHGF) diet versus a processed, extruded dry kibble (EDK) diet. Our findings demonstrate that canine epigenetic ageing clocks provide a practical and quantifiable tool for investigating factors that influence canine ageing and offer preliminary evidence that dietary intervention may affect canine ageing.

## MATERIALS AND METHODS

### Subjects used for epigenetic clock development and validation

A total of 104 privately owned companion dogs between 0.5 and 17 years of age were independently recruited for the development (*n* = 37) and validation (*n* = 67) of the canine buccal DNA methylation‐based epigenetic ageing clock. These dogs were recruited separately from the dietary intervention study and were not included in the feeding trial described below. Dogs were considered healthy based on pet owner‐reported health status and age at the time of enrollment. An independent external validation cohort of 48 companion dogs was subsequently recruited to further evaluate the performance of the finalised epigenetic ageing clock.

### Subjects used for diet intervention trials

Privately owned or rescued‐then‐adopted healthy adult pugs between the ages of 1 and 10 years were recruited for the study. At the time of enrollment, all the dogs were fed commercially available EDK diets, constituting most of their daily caloric intake (≥90% of daily calories). Dogs were considered healthy based on a review of medical records, haematology, serum chemistry and urinalysis obtained from the veterinary clinics where they received primary care or from an intake examination after being rescued. Dogs were excluded if they had a preexisting illness, had been administered medication (except for monthly parasiticides), failed to obtain at least 90% of their daily caloric intake from the assigned study diet, or if the pet owner failed to return one or more buccal mucosal swabs. Pet owner consent forms were obtained prior to study enrollment.

Thirty‐eight dogs were initially enrolled in the study and were divided equally between the MPHGF and EDK diet groups. Twelve participants were disqualified due to failure to return one or more cheek swabs, and one participant was disqualified due to owner non‐compliance for adhering to the study diet. Twenty‐five dogs ultimately completed the 6‐month trial: 18 dogs from the MPHGF diet group and seven from the EDK diet group. Summary statistics are presented in Table 1.

**TABLE 1:** Characteristics of dogs recruited for this study in terms of their sex and diet assignment.

Category	Characteristic	Completed trial	Sex	Age, mean ± SD
Diet assignment	MPHGF	18	M, 8	2.7 ± 1.4
			F, 10	
	EDK	7	M, 2	2.8 ± 1.2
			F, 5	

Abbreviations: EDK, extruded dry kibble; MPHGF, minimally processed, human‐grade food.

### Diets

The MPHGF diet was comprised of a commercially available food formulated by a board‐certified veterinary nutritionist that included cooked, fresh ingredients. The study diet was prepared in industrial batch cooking vessels (Blentech) via conductive thermal processing and utilise freezing as the preservation method and supplied to participants in a frozen state and defrosted prior to feeding. The MPHGF passed a maintenance feeding trial as defined by the Association of American Feed Control Officials. The ingredient list and nutrient profile of the MPHGF diet can be found in Table 2. A board‐certified veterinary nutritionist used the daily metabolisable energy requirement for adult dog maintenance calculation published in the National Research Council's Nutrient Requirements of Dogs and Cats to establish the initial feeding amount of the MPHGF diet.^28^ The dogs were weighed 2‒3 weeks by their owners after starting the MPHGF diet to either maintain or adjust feeding amounts to prevent weight gain or loss. The processed EDK diet and the amount fed to each dog were maintained at the same levels before the study. All dogs had to consume their diet for 90% or more of their daily caloric intake from the assigned study diet throughout the duration of the study. The dogs who were kept on their original EDK consumed a variety of diets, including the following brands: Authority, Royal Canin, Blue Buffalo, Hill's Science Diet and IAMS.

**TABLE 2:** Fresh‐food, human‐grade diet nutrient profile.

Nutrients	Nutrient content per 1000 kcal ME
Crude protein (g)	72
Crude fat (g)	26
Crude fibre (g)	1.4
Carbohydrate (by difference) (g)	100
EPA and DHA (g)	0.17
Calcium (g)	3.4
Phosphorus (g)	2.7
Vitamin D (IU)	144
Vitamin E (IU)	15
Vitamin A (IU)	8287
Vitamin B12 (mg)	0.011
Folate (mg)	0.21
Zinc (mg)	39
Omega 6:3 ratio	10:1
Me (calc) (kcal/kg)	1535

*Note*: MPHGF ingredient list—chicken, rice, spinach, carrots, apples, chicken gizzard, chicken liver, fish oil, dicalcium phosphate, calcium carbonate, salt, choline bitartrate, potassium iodide, zinc amino acid chelate, magnesium amino acid chelate, vitamin E supplement, ferrous amino acid chelate, copper amino acid chelate, cholecalciferol (source of vitamin D3), d‐calcium pantothenate, riboflavin and vitamin B12 supplement.

Abbreviations: DHA, docosahexaenoic acid; EPA, eicosapentaenoic acid; IU, international units; ME, metabolisable energy.

### Sample collection and processing

Buccal cell samples were collected from each dog at designed timepoints. Sample collection was performed by pet owners at home using standardised collection kits provided by the study team, along with detailed written instructions to ensure consistency. Following collection, swabs were immediately placed into DNA stabilisation buffer (Cat. No. R1150, DNA/RNA Shield, Zymo Research) to preserve sample integrity at ambient temperature during storage and transport. The samples were then returned to the Clinical Laboratory Improvement Amendments‐certified facility for extraction and processing. Genomic DNA was purified from cells using the Quick‐DNA Magbead Plus Kit (Cat. No. D4068, Zymo Research) and extracted DNA quality was assessed using a Nanodrop. Bisulphite conversion and library preparation of the DNA for next‐generation sequencing (NGS) were then performed to quantify epigenetic age. A paper‐based questionnaire was completed by pet owners at each collection interval, which included the dog's age, weight and diet.

### Epigenetic age prediction

Dog buccal DNA was bisulphite‐converted using the EZ DNA Methylation‐Lightning Kit (Zymo Research) on the KingFisher Apex (ThermoFisher Scientific). Bisulphite‐converted DNA, containing more than 250 age‐associated CpG loci (genomic regions where a cytosine [C] nucleotide is immediately followed by a guanine nucleotide and where DNA methylation most commonly occurs in mammals), was prepared for the Simplified Whole‐Panel Amplification Reaction Method multiplex platform, which includes a fully automated protocol on the Tecan Fluent (Tecan). The targeted libraries were normalised and pooled to prepare for loading on the NovaSeq X (Illumina), where each specific CpG locus was sequenced at more than 1000× coverage. Sequencing reads were identified using a base calling software and then aligned to the CanFam3 genome using Bismark, an aligner optimised for bisulphite‐converted sequences.^29^ Methylation levels for each ‘C’ were calculated by dividing the number of reads reporting a ‘C’ by the number of reads reporting a ‘C’ or ‘thymine (T)’. The methylation level of 170 age‐associated CpG loci was used for epigenetic age prediction using Zymo Research's proprietary canine DNAge predictor built with the elastic net algorithm of the glmnet R package (version 4.3.1, R Core Team, 2023).^30^

A penalised regression model's coefficients *b*_0_, *b*_1_, …, *b_n_* was used to calculate the transformed age as described in Equation (1):

(1) $$F\left(\mathrm{chronological}\mathrm{age}\right)=b_{0}+b_{1}\mathrm{Cp}G_{1}+\cdots +b_{n}\mathrm{Cp}G_{n}+\mathrm{error}$$

Epigenetic age was estimated as described in Equation (2):

(2) $$\mathrm{Epigenetic}\mathrm{age}=\mathrm{inverse}\;F(b_{0}+b_{1}\mathrm{Cp}G_{1}+\cdots +b_{n}\mathrm{Cp}G_{n})$$

Delta age was calculated as described in Equation (3):

(3) Deltaepigeneticage=epigeneticage−chronologicalage

### Statistical analysis

Statistical analysis of delta age at M0 (baseline) and M6 (month 6) was performed using Wilcoxon signed‐rank test, the wilcox.test function with paired = TRUE in R. A significant cutoff of *p*‐value less than 0.05 was used. Permutation test was performed to adjust sex as a covariante, using the lmp function of the lmPerm package in R. Fisher's exact test, the fisher.test function in R, was conducted to analyse the numbers of dogs whose delta age being younger or older at M6. Analyses were conducted in R (version 4.3.1, R Core Team, 2023). All the data are represented as mean ± standard deviation unless otherwise specified.

## RESULTS

### Validation of buccal swabs as a viable sample source for epigenetic age prediction in companion dogs

**FIGURE 1:**
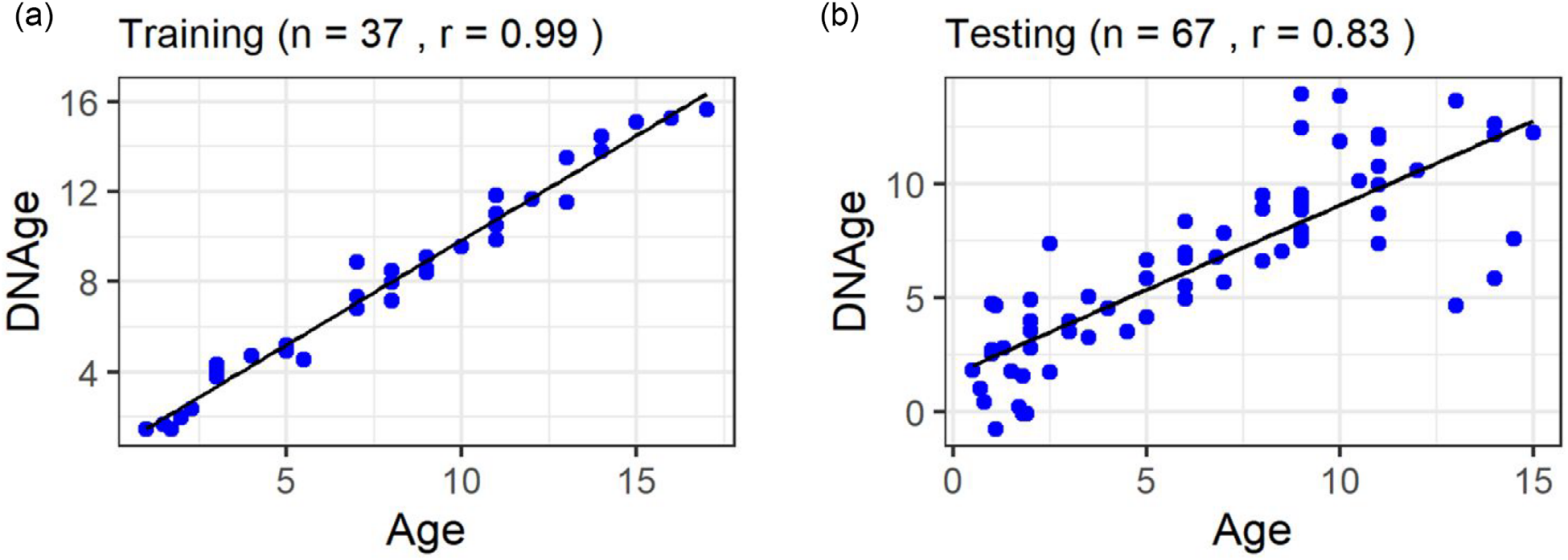
DNA methylation analysis using the proprietary DNAge algorithm to train the program using a training sample data set (a) and to predict the age in an experimental data set (b).

Based on retrospective data analysis, a list of individual CpGs in the dog genome associated with ageing and age‐related diseases has been previously determined from blood.^24^, ^25^, ^31^ To determine whether these age‐associated CpG markers could be applied to a non‐invasive sample source, we evaluated the performance of a canine epigenetic ageing clock using buccal swab DNA. The targeted, age‐related cytosine residues (CpGs) in each DNA sample from these dogs were analysed using targeted bisulphite NGS. DNA methylation patterns resulting from 37 domestic dog samples between the ages of 1.5 and 17 years were used to optimise the canine epigenetic age model for the buccal cell DNA input. The resulting buccal epigenetic ageing clock was then evaluated in a separate testing set of 67 buccal swab samples from dogs ranging from 0.5 to 15 years of age. The model accurately predicted dog epigenetic age, as observed by a correlation to chronological age of 0.99 in the training set (Figure 1A) and a coefficient of determination of 0.83 in the testing set (Figure 1B). To further demonstrate utility, the buccal cell‐based epigenetic ageing clock was validated in an additional independent cohort of self‐reported healthy dogs (Figure S1).

### Epigenetic ageing declined following a 6‐month intervention with MPHGF diet

**FIGURE 2:**
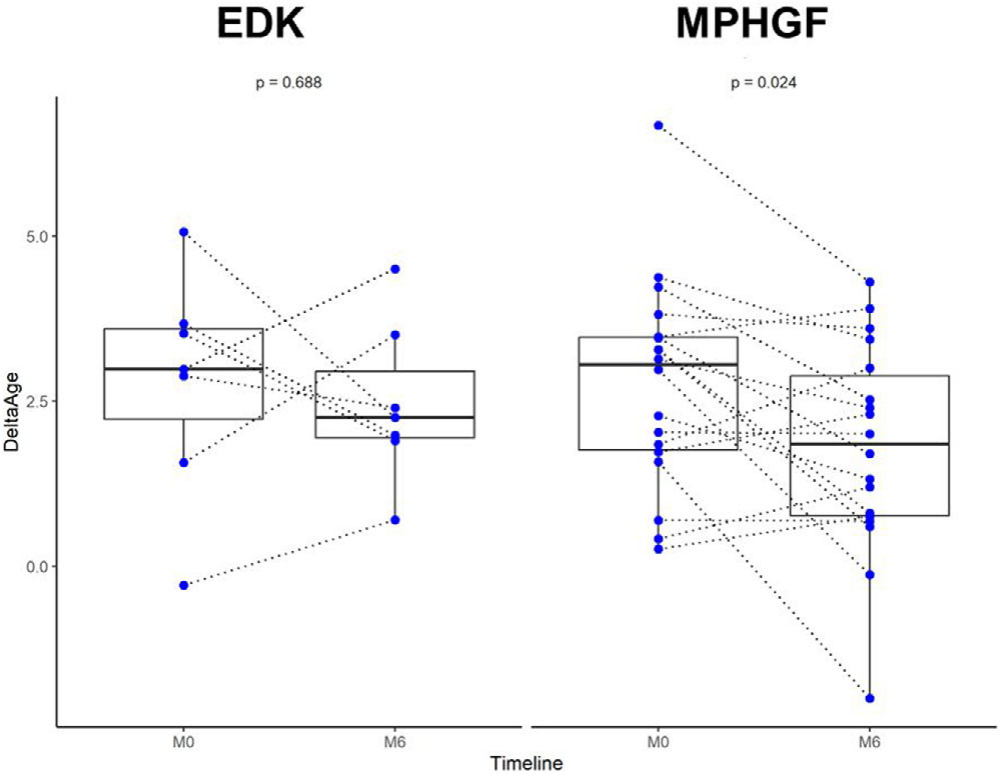
Epigenetic age changes over timepoints (M0 = baseline and M6 = 6 months after enrollment) were assessed to compare delta epigenetic ageing changes between the extruded dry kibble (EDK, left) and the minimally processed, human‐grade food (MPHGF, right). A significant reduced delta epigenetic age was observed in MPHGF diet using Wilcoxon signed‐rank test.

The epigenetic age of dogs was determined and compared for two distinct diet intervention groups tested at timepoints indicated in Figure 2. Dogs fed the MPHGF diet demonstrated a significant reduction in epigenetic ageing pre‐diet (M0) (2.75 ± 1.6 years) versus post‐diet intervention (M6) (1.80 ± 1.6 years; *p* = 0.02). In contrast, dogs fed the EDK diet did not exhibit similar trends in epigenetic ageing to the MPHGF cohort (M0) (2.77 ± 1.7 years) versus post‐intervention (M6) (2.46 ± 1.2 years; *p* = 0.86). There was no significant difference between the two diet groups at the baseline (Table 1).

The result of Fisher's exact test showed a trend that dogs in the MPHGF group were nearly two times more likely to age less fast than those in the EDK control group (odds ratio = 1.90, 95% confidence interval [CI]: 0.28 to Inf, *p* = 0.39), consistent with the Wilcoxon test above. Also, sex‐stratified analysis was performed among female dogs using the Wilcoxon signed‐rank test, the effect of MPHGF in females was still significant (*p* = 0.032, 95% CI: ‒Inf to ‒0.02, median ‒0.97), while that of EDK was insignificant in females (*p* = 0.313, 95% CI: ‒Inf to 1.52, median ‒0.48) (Supporting Information S2).

### Dogs fed MPHGF diet demonstrated significant reductions in DNA methylation status of the KISS1R gene

**FIGURE 3:**
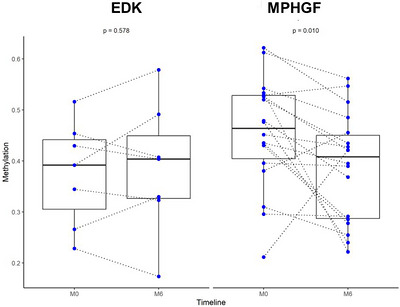
DNA methylation levels for KISS1R gene loci at baseline (M0) and at 6 months after enrollment (M6) for the extruded dry kibble (EDK) diet group (left) and minimally processed, human‐grade food (MPHGF) diet group (right).

Differential DNA methylation levels in the KISS1R gene locus have been previously associated with dog obesity.^18^ The DNA methylation levels at specific KISS1R loci of dogs in the present diet intervention trial were tested by targeted bisulphite sequencing at baseline and 6‐month timepoints (Figure 3) to compare any influence from MPHGF and EDK diets. Results indicate a significant reduction in KISS1R methylation exhibited by the MPHGF diet group comparing M6 to M0 (*p* = 0.01), but there were no significant changes in DNA methylation in test subjects that fed the EDK diet (*p* = 0.578).

### Impact of sex on anti‐ageing in dogs fed MPHGF diets

**FIGURE 4:**
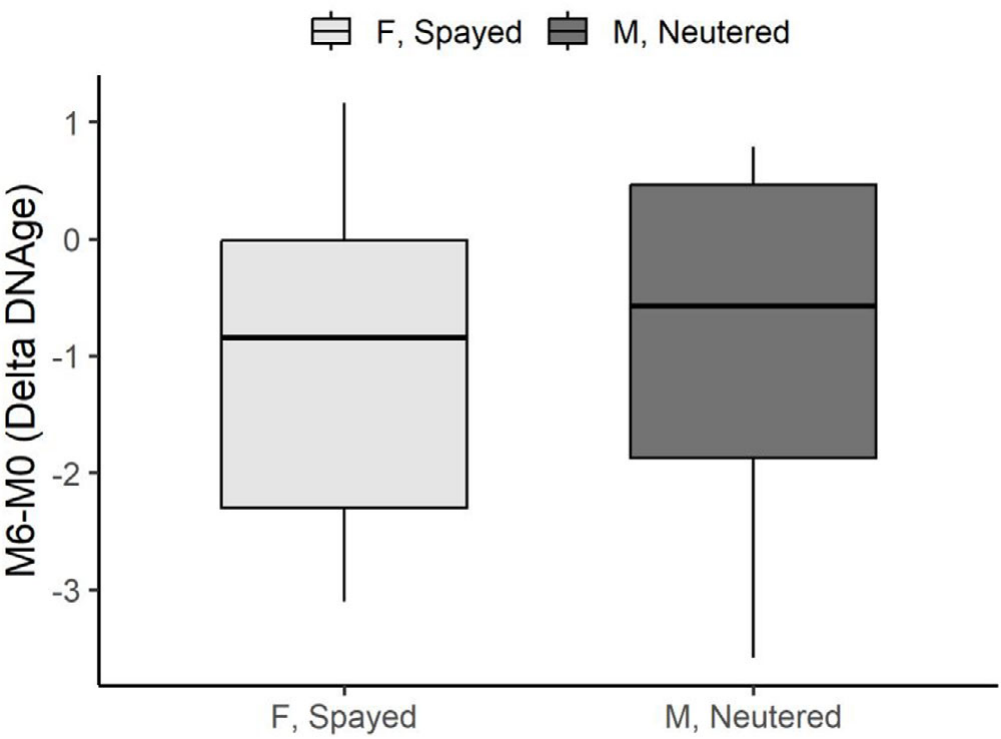
Delta epigenetic age changes 6 months after enrollment (M6) for female (spayed) dogs and male (neutered) dogs in minimally processed, human‐grade food (MPHGF) diet group.

In humans, females tend to exhibit slower epigenetic ageing than males.^25^ The impact of sex on the canine subjects in this study was considered. In the MPHGF diet group, the mean delta epigenetic age reduction (delta epigenetic age M6 minus delta epigenetic age M0) was ‒1.25 in female, spayed participant dogs, compared to ‒0.87 in male, neutered dogs; difference was not statistically significant (*p* = 0.46; Figure 4). These results indicate that sex did not play a significant role in epigenetic age reduction in this study.

## DISCUSSION

This study is first to examine how DNA methylation and a novel epigenetic ageing clock based on buccal cell DNA can be used to test the effects of minimally processed and processed diets on epigenetic ageing in domesticated canines living in human households. The findings demonstrate a trend that MPHGF diet may be more effective than EDK diet in reducing epigenetic ageing in the canines in this study. Additionally, the data indicate that feeding dogs a minimally processed diet may be associated with obesity risk in dogs. Furthermore, this study highlights the potential of epigenetic ageing clocks based on DNA methylation status at specific loci as a practical and efficient tool for evaluating factors that could affect epigenetic ageing and, in turn, canine wellness and lifespan.

From this study, dogs fed with MPHGF diet exhibited a reduction in epigenetic age after 6 months, whereas no significant changes were observed in those test subjects on a processed, EDK diet. These findings collaborate with prior research suggesting that highly processed diets may accelerate ageing compared to fresh‐food diets.^32^, ^33^, ^34^ While longer‐term investigations with additional cohorts are warranted, the results here suggest preliminarily that dietary interventions could play a meaningful role in the canine chronological and biological ageing process particularly by slowing epigenic ageing.

Also noteworthy were the differences observed in DNA methylation status at the KISS1R gene locus between diet groups.^18^ Dogs in the MPHGF group exhibited a reduction in KISS1R methylation levels after 6 months, whereas those consuming EDK diet showed elevated levels. In mammals, KISS1R has been associated with longevity, potentially through its role in metabolic regulation and obesity.^35^ It is interesting to note that in the present study, bodyweight remained stable across both groups of test subjects, suggesting that dietary composition and potentially metabolic and other processes, rather than weight fluctuations, influenced the levels of KISS1R methylation. Future research should continue to explore the relationship between diet, metabolism, bodyweight and KISS1R methylation in canine obesity and ageing to clarify the molecular mechanisms underlying these associations.

This study also underscores the practicality of using a sample‐specific (oral swab) epigenetic ageing clock to facilitate a new era of molecular tools available to veterinary researchers to investigate ageing. The epigenetic ageing clock offers a time‐ and cost‐effective alternative to traditional methods when conducting longitudinal ageing studies.^36^ In addition, compared to traditional ageing models constructed using purebred‐based training and testing, the epigenetic ageing clock developed in the present study was derived using dogs with genetically diverse backgrounds, regardless of breed or size.^26^ Also implementing a novel machine learning approach, we successfully validated this epigenetic ageing clock with the test set of canine subjects. For broad adoption and test subject recruitment in the future, easy collection of non‐invasive cheek swab samples allows for convenient, at‐home sampling by pet owners. As similar epigenetic ageing measures have been successfully deployed in other mammalian species (e.g., humans and mice), their adoption in veterinary research could provide valuable insights into canine health span and longevity.^37^, ^38^

As a pilot study, several limitations should be considered when interpreting the present findings. It is important to emphasise that the current study focused on comparing the effects of minimally processed versus processed diets on epigenetic ageing in dogs, and that the processed diets were sourced from multiple vendors. Future studies could stratify diets based on commercial providers and ingredient composition to elucidate specific influences on epigenetic ageing. Furthermore, the diet study population consisted uniformly of a single breed of dog (i.e., pug), which could have ramifications to the data versus conducting the studies with other breeds of dog. However, recent research on this topic suggests that DNA methylation patterns exhibit minimal differences between dog breeds, indicating that the observed epigenetic effects demonstrated in this study may be broadly applicable.^39^ Different living environments, including housing conditions and pet owner's lifestyle, could also introduce variability and environmental stress that can affect the epigenetic ageing process in privately owned dogs. Furthermore, discrepancies in sample sizes between diet groups could have influence on this study's outcomes. Although a balanced number of dogs were recruited for the two different diet groups at the beginning of trial, a few dogs dropped out of the EDK group due to lack of compliance with the study's protocol and guidelines. The Wilcoxon signed‐rank test is a non‐parametric method that remains valid under small group sizes because it avoids strict parametric assumptions (such as normality); however, it does not compensate for the reduced statistical power associated with small sample sizes. Therefore, the limited number of dogs in the EDK group should be considered when interpreting the study findings. A larger, adequately powered validation study with improved participant retention is planned to further evaluate and confirm current observations. Other considerations include potential discrepancies in the bodyweights reported by pet owners rather than determined in a controlled setting. The possibility of unreported weight fluctuations could impact KISS1R methylation. Future studies should endeavour for incorporating more standardised dietary formulations, balanced sample sizes and controlled environmental factors to further test specific contributions to differential DNA methylation linked to KISS1R and epigenetic ageing.

## CONCLUSIONS

The results of the present study demonstrate evidence that a minimally processed, human‐grade diet can influence epigenetic age progression in canines. These findings highlight the importance of dietary interventions as a key contributor in regulating epigenetic ageing. The tools and methods developed and highlighted herein should pave the way for continued molecular‐based research into canine health span and longevity and may also facilitate the development of similar non‐invasive epigenetic ageing approaches in other domestic animal species.

## AUTHOR CONTRIBUTIONS

*Conceptualisation, methodology, investigation and project administration*: Vincent Michels, Dan Su and Xiaojing Yang. *Investigation and data curation*: Aimee Chen. *Formal analysis, software and visualisation*: Wei Guo. *Writing—original draft*: Xiaojing Yang and Janina A. Krumbeck. *Writing—review and editing*: Vincent Michels, Dan Su, Aimee Chen, Wei Guo, Xiaojing Yang and Janina A. Krumbeck. All the authors have read and approved the final manuscript.

## CONFLICTS OF INTEREST

The authors declare they have no conflicts of interest. Dan Su and Vincent Michels are employees of JustFoodforDogs who funded the study.

## ETHICS STATEMENT

All procedures in this study were non‐invasive and involved buccal swab sample collection performed by pet owners at home. Written informed consent was obtained from all pet owners prior to participation. The study was conducted in accordance with ethical principles for research involving privately owned companion animals, and no procedures were expected to cause pain, harm, or distress to the animals.

## INFORMED CONSENT STATEMENT

Written informed consent was obtained from all pet owners prior to study enrollment.

## Supporting information



Supporting Information

## Data Availability

The raw sequencing data that support the findings of this study are available from the corresponding author upon reasonable request.
