# Supplementary material for: Minimally processed, human‐grade diet alters canine epigenetic ageing: Results from a pilot study
Source: Vet Rec Open. 2026 Aug 5;13(2):e70047. doi: 10.1002/vro2.70047 (PMC13442920; doi:10.1002/vro2.70047)
Supplement: Supplementary file 1 [file VRO2-13-e70047-s001.docx]

Figure S1. **Validation of mydog DNAge® epigenetic clock in an independent cohort of health dogs.**

**Figure S1:** DNA methylation analysis using the proprietary DNAge® algorithm to predict the DNAge in an independent cohort of dogs.

S2. **Supplementary Statistical Analysis: Method and Results**

**Methods:**

Because dogs entered the study at different chronological ages, direct comparison of raw epigenetic age values could be confounded by baseline age differences. Therefore, delta epigenetic age (ΔAge) was calculated for each dog as:

ΔAge = Epigenetic Age − Chronological Age

This metric reflects a dog's Epigenetic age acceleration compared to its chronological age.

To evaluate the effect of dietary intervention, ΔAge was determined at baseline (M0) and after six months of dietary intervention (M6). Changes in ΔAge were then assessed within each dog, allowing each animal to serve as its own control and enabling comparison of biological aging trajectories independent of age at enrollment.

For categorical analysis, dogs were classified as "Younger" if ΔAge at M6 was lower than ΔAge at M0, indicating a reduction in epigenetic age relative to chronological age during the study period. Dogs were classified as "Older" if ΔAge at M6 was equal to or greater than ΔAge at M0. The numbers of dogs classified as Younger or Older were compared between dietary groups using Fisher's exact test.

To explore the potential influence of sex on the observed outcomes, an additional sex-stratified analysis was performed among female dogs using the Wilcoxon signed-rank test to compare paired ΔAge values between M0 and M6. Male dogs in the EDK group were not analyzed separately due to the limited sample size (n = 2).

The original statistical outputs are provided below.

| Group | DAge_M0 | DAge_M6 | Sex | Younger at M6 compared to M0 |
| --- | --- | --- | --- | --- |
| control | 1.5667 | 3.5 | M | Not |
| control | 2.98 | 4.5 | F | Not |
| control | 2.88 | 2.4 | F | Yes |
| control | 3.67 | 1.9834 | M | Yes |
| control | 3.52 | 1.9 | F | Yes |
| control | -0.29 | 0.7 | F | Not |
| control | 5.0634 | 2.25 | F | Yes |
| treatment | 3.81 | 3.6 | M | Yes |
| treatment | 1.58 | -2 | M | Yes |
| treatment | 0.41 | 1.2 | M | Not |
| treatment | 3.13 | 2.4 | F | Yes |
| treatment | 3.273 | 0.6 | F | Yes |
| treatment | 1.84 | 3 | F | Not |
| treatment | 3.45 | 1.7 | F | Yes |
| treatment | 4.227 | 2.52 | M | Yes |
| treatment | 4.374 | 3.434 | M | Yes |
| treatment | 2.277 | 1.3167 | F | Yes |
| treatment | 3.28 | 0.8 | F | Yes |
| treatment | 3.47 | 3.9 | M | Not |
| treatment | 0.26 | 0.75 | F | Not |
| treatment | 2.97 | -0.13 | F | Yes |
| treatment | 6.67 | 4.3 | M | Yes |
| treatment | 0.6934 | 0.6834 | F | Yes |
| treatment | 1.73 | 2.3 | M | Not |
| treatment | 2.03 | 2 | F | Yes |

**Results:**

1. **Fisher’s exact test of becoming younger at M6 between MPHGF (treatment) and EDK (control) groups.**

Input data of the numbers of dogs becoming younger or older at M6 compared to M0:

| Group | Younger | Older |
| --- | --- | --- |
| MPHGF | 13 | 5 |
| EDK | 4 | 3 |

Fisher's exact test:

[1] "Contingency Table:"

Outcome

Group	Younger Older

MPHGF	13	5

EDK	4	3

Fisher's Exact Test for Count Data

data: data_matrix

p-value = 0.3931

alternative hypothesis: true odds ratio is greater than 1

95 percent confidence interval:

0.2786497	Inf

sample estimates:

odds ratio

1.895518

2. **Wilcoxon signed-rank test of age change of females only in MPHGF (treatment) and EDK (control).** **To explore the potential influence of sex, a stratified analysis was performed among female dogs.**

| Group | DAge_M0 | DAge_M6 | Sex |
| --- | --- | --- | --- |
| control | 2.98 | 4.5 | F |
| control | 2.88 | 2.4 | F |
| control | 3.52 | 1.9 | F |
| control | -0.29 | 0.7 | F |
| control | 5.0634 | 2.25 | F |
| treatment | 3.13 | 2.4 | F |
| treatment | 3.273 | 0.6 | F |
| treatment | 1.84 | 3 | F |
| treatment | 3.45 | 1.7 | F |
| treatment | 2.277 | 1.3167 | F |
| treatment | 3.28 | 0.8 | F |
| treatment | 0.26 | 0.75 | F |
| treatment | 2.97 | -0.13 | F |
| treatment | 0.6934 | 0.6834 | F |
| treatment | 2.03 | 2 | F |

Wilcoxon signed rank exact test

data: df_females$DAge_M6[df_females$Group == "treatment"] and df_females$DAge_M0[df_females$Group == "treatment"]

V = 9, p-value = 0.03223

alternative hypothesis: true location shift is less than 0

95 percent confidence interval:

-Inf -0.02

sample estimates:

(pseudo)median

-0.97

Wilcoxon signed rank exact test

data: df_females$DAge_M6[df_females$Group == "control"] and df_females$DAge_M0[df_females$Group == "control"]

V = 5, p-value = 0.3125

alternative hypothesis: true location shift is less than 0

95 percent confidence interval:

-Inf 1.52

sample estimates:

(pseudo)median

-0.48
